# Research progress on the pathogenesis of diabetic retinopathy

**DOI:** 10.1186/s12886-023-03118-6

**Published:** 2023-09-11

**Authors:** Hongbo Li, Xinyu Liu, Hua Zhong, Jiani Fang, Xiaonan Li, Rui Shi, Qi Yu

**Affiliations:** 1 School of Basic Medical Sciences, Xi’an Medical University, Xi’an, China; 2 Department of Ophthalmology, Shaanxi Provincial People’s Hospital, Xi’an, China; 3 Institute of Basic and Translational Medicine, Xi’an Medical University, Xi’an, China

**Keywords:** Diabetic retinopathy, Oxidative stress, Inflammatory reaction, Mitochondrial disorders, Gene epigenetic inheritance, Hemoglobin, Intestinal flora, Amino acid metabolism

## Abstract

Diabetic retinopathy is one of the most common and serious microvascular complications of diabetes mellitus. There are many factors leading to diabetic retinopathy, and its pathogenesis is still unclear. At present, there are still no effective measures for the early treatment of diabetic retinopathy, and the treatment options available when diabetes progresses to advanced stages are very limited, and the treatment results are often unsatisfactory. Detailed studies on the molecular mechanisms of diabetic retinopathy pathogenesis and the development of new therapeutic agents are of great importance. This review describes the potential pathogenesis of diabetic retinopathy for experimental studies and clinical practice.

## Introduction

Diabetic retinopathy (DR) is one of the common complications of type 2 diabetes mellitus (T2DM) and is an irreversible and blinding eye disease. Depending on the degree of progression, DR is categorized as non-proliferative diabetic retinopathy(NPDR) and proliferative diabetic retinopathy(PDR), specifically mild non-proliferative diabetic retinopathy, moderate non-proliferative diabetic retinopathy, severe non-proliferative diabetic retinopathy and proliferative diabetic retinopathy. In NPDR, the condition is accompanied by loss of perivascular cells in the retina, leakage of dilated microvessels, and formation of small aneurysms until the retina swells and affects the patient's vision. In PDR, the blood-retinal barrier is broken and the vascular perfusion pressure is reduced. The most typical feature of PDR is the proliferation of the ischemia-mediated imbalance between angiogenic and antiangiogenic factors leading to neointimal hyperplasia, which can lead to vitreous hemorrhage and retinal detachment. Vision loss is primarily associated with two advanced diseases: diabetic macular oedema(DMO) and PDR; DMO can occur at any stage of diabetic retinopathy, but the risk increases with the severity of the DR lesion, with a prevalence of up to 71% of patients with PDR [1]. Hyperglycemia and nuclear factor-kappaB (NF-κB) hypertension are the main risk factors for DMO, which primarily destroys the blood-retinal barrier by altering endothelial growth factor (VEGF) and pro-inflammatory cytokines pro- inflammatory cytokines, which can lead to vascular leakage and ultimately to blindness [2]. This article provides a review and outlook of the main influencing factors and pathogenesis involved in DR-related research areas and potential therapeutic targets.

### Oxidative stress

Oxidative stress (OS) is an adverse phenomenon caused by the imbalance between the formation and elimination of oxygen free radicals, which plays an important role in the development of DR. Studies have shown that diabetic patients have increased levels of reactive oxygen species (ROS) in hyperglycemia (HG) and decreased levels of antioxidants such as superoxide dismutase (SOD) and glutathione (GSH). This leads to an imbalance in the oxidation-antioxidation system, which exposes retinal tissues and cells to ROS attack and accelerates apoptosis and DR pathology [3]. Therefore, the elevated level of ROS can be used as an important reference indicator in DR lesions.

Current research related to OS-induced DR is more extensive and involves numerous mechanisms. Studies have shown that the HG environment activates polyol pathways that have multiple effects on the generation of oxidative stress in DR. Catalyzed by aldose reductase, excess glucose uses nicotinamide adenine dinucleotide phosphate (NADPH) as an electron donor to form sorbitol, which is strongly hydrophilic and loses its lipid membrane penetration ability, increasing cell osmotic pressure and leading to retinal capillary permeability damage. In addition, sorbitol is reduced to fructose by the action of sorbitol dehydrogenase, which further depletes a large amount of NADPH, leading to a decrease in cofactors for GSH synthesis and a weakened resistance to oxidative stress, accelerating the damage of retinal pigment epithelial cells by ROS [4]. Under a prolonged HG environment, reducing sugars react with proteins, lipids, and other macromolecular substances at their active sites to form stable glycosylation end products after a complex series of chemical rearrangements, which promote NF-κB activation by binding to late glycosylation end product receptors, leading to apoptosis and increased vascular endothelial permeability in pericytes, as well as activating downstream NADPH oxidase to accelerate the use of NADPH to increase ROS production [5]. Studies have shown that high blood glucose levels as well as severe insulin resistance and obesity activate high NF-kB expression in retinal vascular cells and increase retinal inflammation and oxidative stress, but the latter two do not exhibit DR-specific vasculopathy, and that diabetes or hyperglycemia activates pathways such as NF-κB, inflammation, or oxidative stress leading to retinal capillary dysfunction, which indirectly suggests that hyperglycemia plays a crucial role in the progression of DR [6]. The NOX family of proteins is an important member of the NADPH oxidase family and includes multiple isoforms that play different roles in mediating ROS or in the retina. Related studies in DR have focused on NOX1, NOX2, and NOX4. Studies have shown that excessive ROS production by NOX2/NADPH in HG increased arginase-1 expression and decreased the utilization of NO, which damages the vascular repair mechanism and causes retinal vascular leakage [7]. Researchers studied inducible human NOX5 transgenic mice in an oxygen-induced retinopathy model by in vitro and in vivo experiments and found that NOX5 significantly induced oxidative stress and elevated VEGF and intercellular adhesion factor levels, which in turn exacerbated neovascularization and vascular infiltration and increased damage to the visual system, and it was also demonstrated that inhibition of NOX1/4 expression and NOX5-siRNA reduced high glucose-induced oxidative stress, angiogenesis, and upregulation of inflammatory factors [8]. In addition, the production of excess reactive oxygen species is inextricably linked to the occurrence of lipid peroxidation. Oxygen-derived free radicals, such as hydroxyl and hydroperoxides, have been shown to oxidize phospholipids and other lipid components in cell membranes, causing lipid peroxidation, which in turn generates ROS promoting senescence of retinal pigment epithelial cells, and the peroxidation products can facilitate the process of permeation of ROS from the mitochondria, which also suggests that cutting off the pathway of origin of peroxides by inhibiting the onset of lipid peroxidation plays an important role in the control of DR This also suggests that cutting off the source pathway of peroxides by inhibiting the occurrence of lipid peroxidation is important for the control of DR [9]. Fatty acid binding protein 4 (FABP4) is a free fatty acid (FFA) chaperone protein that plays an important role in maintaining glucose and lipid homeostasis. Studies have shown that the abnormally high expression of serum FABP4 in patients with DR results in a disturbed balance between glucose and lipid metabolism, exacerbating the development of DR, and FABP4 inhibitors can attenuate lipid peroxidation and oxidative stress, effectively reducing retinal damage in DR. Meanwhile, the degree of FABP4 elevation was positively correlated with the lesions, suggesting that FABP4 can be used as a prognostic marker for DR patients and an important target for inhibiting lipid peroxidation to delay retinal damage [10].

The current clinical debate on antioxidant stress injury revolves around the increase of antioxidant enzyme content and activity. Normally, Nrf2 is located in the cytoplasm linked to a redox-sensitive protein (kelch-like ECH-associated protein 1, Keap1) and the ubiquitin ligase Cullin3. Under oxidative stress, it can induce conformational modification of Keap1 cysteine residues, transportation of Nrf2 and combination with the antioxidant reaction elements (ARE), which lead to the expression of antioxidant genes to maintain cell homeostasis [11]. In the streptozotocin-administered diabetic mouse model, the increased expression of Keap1 and insufficient translocation of Nrf2 to the nucleus resulted in decreased activity of Nrf2-associated antioxidant enzymes, such as heme oxygenase 1, NADPH dehydrogenase, thioredoxin reductase, catalase, and glutathione reductase, which reduced the antioxidant capacity of the body and aggravated DR [12]. The polymorphism analysis of Nrf2 gene locus and the regulation of its expression levels in and out of the nucleus may become a new prevention and control strategy for DR, but a lot of research is still needed on how to accurately screen gene loci and analyze the correlation between loci. Bardoxolone methyl (BM), considered the most important synthetic triterpenoid. BM can effectively increase estimated glomerular filtration rate (eGFR) in clinical trials, showing positive therapeutic effects in chronic kidney disease (CKD). Meanwhile, BM is an oral antioxidant and inflammation modulator, which can increase the antioxidant capacity by activating the Nrf2- Keap1-ARE signaling pathway, and slow down the development of inflammation by inhibiting the NF-κB signaling pathway, which may directly intervene in the development of diabetic kidney disease (DKD) and DR [13].

### Inflammatory reaction

Inflammation plays a key role in the pathogenesis of DR. The measurement of the expression of inflammatory factors can be used as an important indicator for the early diagnosis and prognosis of DR. The levels of interleukin-1β (IL-1β), IL-6, IL-8, IL-17A and tumor necrosis factor-α (TNF-α) were found to be significantly elevated in DR patients, and the expression was positively correlated with the severity of the disease [14]. In HG, the expression of TNF-α, monocyte chemoattractant protein-1, IL-1β and other inflammatory factors is up-regulated [15]. TNF-α induces the expression of Runt-related transcription factor 1 in human retinal capillary endothelial cells (HRCECs) by phosphorylating c-Jun N-terminal protein kinase. In turn, it promotes EC proliferation and vascular migration [16]. Increasing serum IL-6 content can cause EC dysfunction, change vascular dynamics, improve vascular permeability, and eventually lead to blood leakage [17]. In addition, chronic inflammation will lead to vascular occlusion and synthesis of new vessels, accelerating the transition to PDR. Studies have shown that nucleotide-binding oligomerization domain-like receptor protein 3 (NLRP3) and IL-1β levels are significantly elevated in the retina of PDR patients and are involved in the formation of late pathological neointima [18]. Thioredoxin-interacting protein (TXNIP) is a protein that promotes oxidative stress and apoptosis, and excessive expression of ROS and TXNIP in a high glucose environment will activate NLPR3 inflammatory vesicles in large numbers and thus activate IL-1β leading to apoptosis of pericytes in the retina [19]. Considering the complexity of the sources that trigger the inflammatory response, most clinical interventions currently adopted are based on the level of early prevention and later disease control. In the HG state, the increase in reactive oxygen radicals plays an important role in the inflammatory response and the subsequent complex oxidative stress response, which in turn causes a vicious circle. Therefore, the combined measurement of inflammatory factors and oxidative stress indicators in serum can provide a clinical reference for the diagnosis and prognosis of DR patients.

### Mitochondrial disorders

#### Mitochondrial DNA disorders

The expression disorder of mitochondrial DNA (mtDNA) plays an important role in DR. Mitochondria are abundant in highly metabolically active photoreceptor cells and retinal pigment epithelium. In mitochondria, high circulating glucose can produce a large amount of ROS by increasing the electron flux of the electron transport chain (ETC) and reducing the specific activity of complex III, which directly damages mitochondrial structure and mtDNA. In addition, the continuous increase of ROS increased the expression of matrix metallopeptidase 2(MMP-2) and MMP-9, which were transferred from the cytoplasm to the mitochondria with the help of heat shock protein 70, destroyed gap junction proteins and led to the exudation of cytochrome C, thereby accelerating capillary apoptosis. This also precedes the appearance of pathological features in DR [20]. Homocysteine levels were found to be more than threefold elevated in the retina of patients with DR and were strongly correlated with mtDNA expression levels [21]. In the HG state, mtDNA is impaired by oxidative stress, base mismatch rates are increased, and transcription of mtDNA-encoded genes is reduced. The increase in homocysteine further exacerbates the extent of damage and reduces the transcription of mtDNA-encoding genes that are essential for maintaining normal ETC function, thereby affecting electron chain transfer and generating a vicious cycle of excess peroxides and free radicals. In addition, homocysteine promotes mitochondrial damage by activating MMP-9 and inhibits the transcriptional activity of Nrf2 impairing the resistance of the mitochondrial defense system to oxidative stress [22]. Thus, high homocysteine levels increase the risk of DR in diabetic patients, which provides a new clinical direction of reference for the early determination of DR disease.

#### Mitochondrial allostery

Mitochondria are highly dynamic, adapting to cellular demands through fusion, division, autophagy and biosynthesis. Under normal conditions, biosynthesis safeguards the replication of mitochondrial genes and proteome, and autophagy selectively degrades damaged or excess mitochondria through lysosomes to prevent apoptosis caused by mitochondrial abnormalities. However, in diabetic mice, mitosis is increased in Müller cells due to impaired mitochondrial transcription factor A downstream of biosynthesis, resulting in reduced mitochondrial numbers [23]. Mitochondrial fission is dominated by dynamin-1-like protein (Drp1), and outer membrane fusion is controlled by fusion protein mitofusin (Mfn), which includes Mfn1 and Mfn2. In DR, the retinal mitochondria are swollen, the expression of Mfn2 is decreased, and the expression of Drp1 is increased, which divides play a dominant role, breaking the dynamic balance and aggravating the damage of mitochondria and increasing the production of ROS [24]. A recent study found that mitochondria-targeting small molecule peptide SS31 can reduce excessive mitochondrial fragmentation in mice by downregulating the expression of Drp1 and increasing the expression of Mfn1 and Mfn2, thus suggesting that SS31 can protect diabetic mice from HG-induced changes in mitochondrial integrity and avoid further subsequent reactions [25].

Mitochondria in diabetic patients are affected by oxidative stress, allostery, DNA damage and other factors. Mitochondria are damaged by oxidative stress and DNA damage. Damaged mitochondria can in turn increase the level of oxidative stress and the degree of DNA damage to change the structure and stability of mitochondria.

### Gene epigenetic inheritance

#### DNA methylation

DNA methyltransferase (DNMT1) is the key enzyme to maintain DNA methylation. Studies have shown that the content of DNMT1 in diabetic retinal cells is significantly increased, and it mainly binds to the D-loop region of mtDNA, which increases the level of 5mC and the degree of methylation, destroys the important genes encoding the ETC system, and leads to a vicious cycle of increased superoxide free radical level [26]. The mtDNA polymerase γ regulatory region CpG site remains hypermethylated blocking the binding of the mismatch repair enzyme POLG to mtDNA, inhibiting the transcriptional efficiency of DNA, causing mitochondrial dysfunction and accelerating retinal capillary apoptosis [27]. At the same time, high levels of histone methylation transferase acting at the NF-κB promoter in the HG state activate its transcriptional expression, which in turn accelerates retinal capillary apoptosis [28]. Decreased methylation levels of mitochondrial DNA and related genes have the potential to increase mitochondrial homeostasis and delay the development of DR. Studies related to histone acetylation have been conducted less frequently, mainly around acetylases. In the retinal cells of diabetic mice, histone deacetylase 6 expression was reduced and the acetylation levels of substrates H3K56 and H3K9 were increased, which prompted the release of VEGF from Müller cells and accelerated the apoptosis of retinalcells [29].

#### Differential expression of ncRNA

Noncoding RNA (ncRNA) represented by microRNAs (miRNAs), long noncoding RNAs (lncRNAs) and circular RNAs (circRNAs) are significantly differentially expressed in DR. They regulate gene expression at the transcriptional and post-transcriptional levels and affect a variety of molecular pathways.

miRNAs are single-stranded ncRNAs consisting of 20–24 nucleotides, which are more stable in tissue fluid and blood. Exosomes are small vesicles that can be secreted by almost all cells and can carry RNA, DNA, lipids, proteins and metabolites. miRNA can interact with the 3′-untranslated region(3′-UTR) of targeted mRNA through its 6 nt seed sequence, and mediate posttranscriptional gene silencing (PTGS) in the cytoplasm [30]. miRNAs play an important role in the induction of DR. Studies have shown that at high glucose levels, the levels of anti-angiogenic miRNAs ( miR-106 a-5 p, miR-20 a-5 p, and miR-20 a-3 p) are reduced, leading to increased expression of VEGF and thus promoting damage to retinal photoreceptors [31]. Some miRNAs can aggravate the progression of PDR in the condition of HG. Overexpressed miR-221 binds to the 3′-UTR of SIRT 1 mRNA and downregulates its expression, blocking the Nrf 2 pathway and increasing the apoptosis of retinal microvascular endothelial cells (RMEC) [32]. The research on targeted RNA in retinal neuropathy focuses on Müller cells. miRNA can interfere with the destruction of Müller cells under HG conditions and play a protective role. For example, up-regulation of miR-486-3P expression can protect Müller cells from apoptosis caused by oxidative stress and inflammatory stimulation under HG conditions [33]. In RMEC exert protective effects by targeting VEGF, for example, miR-15b and miR-152 can directly or indirectly downregulate VEGF expression in RMECs in the HG state and inhibit vascular endothelial cell proliferation [34, 35].

Differential expression of circRNs A in human retinal vascular endothelial cells (HRVECs) has a significant effect on the progression of DR. overexpression of circHIPK3 in HRVEC cytoplasm inhibits miR-30a-3p activity and upregulates expression of VEGFC, FZD4 and WNT2, resulting in increased endothelial proliferation and vascular dysfunction. Silencing circHIPK3 alleviated retinal vascular dysfunction [36]. Therefore, circRNA is a potential target for controlling PDR.

lncRNAs are non-coding protein transcripts consisting of more than 200 nucleotides that regulate gene expression both pre- and post-transcriptionally. lncRNA MALAT1 is an important research target in the direction of DR. MALAT1 promotes glucose-induced angiogenesis and inflammatory responses in HRECs by upregulating endoplasmic reticulum stress, increasing Keap1 transcript levels obstruct the nuclear movement of Nrf2 and inhibit the expression of antioxidant-responsive enzymes to break the retinal microvascular antioxidant defense system [37, 38]. Knockdown of MALAT1 inhibits cell proliferation, migration and angiogenesis of human retinal microvascular endothelial cells (HRMECs) by targeting miR-125b and suppressing the VE-cadherin/β-catenin complex. Inhibition of MALAT1 may serve as a potential target for anti-angiogenic therapy for DR [39].

### Levels of hemoglobin

Anemia is defined as a decrease in hemoglobin concentration, a sign of reduced oxygen-carrying capacity. As one of the complications of diabetes, it adversely affects the progression of diabetes-related microvascular complications, leading to a diminished vasodilatory response and late neointima formation. Numerous clinical studies have found that anemia and hemoglobin levels become independent risk factors for the development and progression of DR. Logistic regression analysis of hemoglobin (Hb) level in patients with type 2 diabetes showed that the Hb level in the DR Group was significantly lower than that in the normal control group, and the incidence of anemia was significantly increased. For every 1 g/dl increase in Hb, the risk of DR decreased by 19%. Low Hb can reduce vascular shear stress, inhibit microvascular remodeling and tension regulation, which in turn leads to retinal tissue damage and promotes the development and deterioration of DR [40]. Hypoxia has an impact on the development of DR. Hypoxia-inducible factor (HIFS) is an important regulator of oxygen homeostasis in the body's response to hypoxia. Currently, the mechanism of HIF-1α's role in DR is complex. HIF-1α plays an unfavorable role as a central stimulator of angiogenesis in PDR, as evidenced by the fact that hypoxia induces the up-regulation of HIF-1α, which in turn increases the expression of VEGF and promotes the formation of retinal blood vessels [41]. Recent studies have shown that HIF-1α overexpression in diabetic rats enhances the invasion, migra tion, and permeability of ARPE-19 cells, exacerbating retinal damage [42]. It is also worth mentioning that patients with DKD and anemia are at a significantly higher risk of developing PDR, with some combination of the two. This is mainly reflected in the estimated glomerular filtration rate (eGFR) and urine albumin-to-creatinine ratio (UACR). Hb may be a mediator of the correlation between UACR and eGFR and the risk of DR. UACR abnormalities affect the risk of DR directly or indirectly by lowering Hb levels, whereas eGFR indirectly affects the risk of DR through decreased Hb levels [43]. Although both are important risk factors for DR, UACR is more strongly associated with DR than eGFR. DKD is not only one of the serious complications of TM, but also contributes to the development of DR. The American Diabetes Association's (ADA) recent "Standards of Medical Care for Diabetes—2020" recommends that effective control of blood pressure, lipid and glucose levels can help reduce the risk or slow the progression of DR, but does not mention Hb [44]. As one of the important items of routine blood tests, Hb has the characteristics of rapid detection and easy access, so the Hb level in patients with T2DM should be taken seriously. Moreover, diabetic patients especially those with a high risk of DKD or anemia should be recommended to have regular testing of early retinal changes to prevent the occurrence of DR. Hb level and anemia rate are decreasing and increasing with the severity of DR, respectively. Controlling Hb levels in patients with DR has a greater clinical significance for disease progression.

### Intestinal flora dysregulation

As an important part of the gastrointestinal tract environment, the change of microecological environment of microbiota often induces many metabolic diseases in humans. Studies have shown that the imbalance of the gastrointestinal ecological environment can lead to the imbalance of microorganisms and their metabolites, promote inflammation, change glucose homeostasis and insulin resistance. At the same time, disturbances in the intestinal flora can affect the fluctuation of related biochemical factors in the body, such as reduced levels of bile acids and endocrine regulatory peptides and increased levels of lipopolysaccharides, which further induce the development of T2DM [45].In addition, it has been confirmed that there are significant differences in viral and fungal communities in the gastrointestinal tract between patients with type 2 diabetes and normal persons [46]. Gut-retinal axis plays an important role in the development of DR. DR Patients have changes in the diversity and abundance of gut microbiota, mainly manifested as a reduction in anti-inflammatory, probiotic and other potentially pathogenic bacteria [47]. Some intestinal bacteria can highlight their anti-inflammatory effects by reducing cytokines and chemokines. For example, ingestion of Lactobacillus paracei can suppress retinal inflammation by decreasing the loss of cytokine-producing macrophages and age-related retinal cells [48]. Although no statistically significant differences have been found in studies of the gut-retinal axis in patients with DM and DR at this time, the gut-retinal axis has been shown to be an important potential target for DM and its associated complications.

### Amino acid metabolism

With the rapid development of metabolomics in recent years, it has been used to identify complex endogenous metabolic phenotypes in various diseases such as diabetes mellitus. Intraocular fluid (vitreous fluid and atrial fluid) can directly reflect metabolic changes in the eye, but considering the limitations of sample acquisition requiring invasive intraocular surgery, there is a rationale for selecting peripheral blood for differential amino acid metabolism analysis. Glutamate and glutamine were found to be significantly different between diabetic and non-diabetic patients and could be used as candidate biomarkers to differentiate DR patients [49]. Glutamine is relatively easily converted to glutamate, which plays an important role in retinal metabolism as a nutritional supplement and excitatory amino acid. Glutamate metabolism is mainly concentrated in retinal ganglion cells (RGCs), endothelial cells and Müller cells. In RGCs, excess amino acids induce increases in intracellular Ca^2+^ levels mainly through massive activation of NMD-type glutamate receptors (NMDAR), causing nNOS (neuronal nitric oxide synthase, nNOS) upregulation, mitochondrial dysfunction, and production of ROS, leading to excitotoxicity which is further physiological disruption of the retina, including oxidative stress, inflammation, and neuronal apoptosis [50]. The same toxic process was observed in the other two cell lines. Notably, plasma metabolite levels of arginine are elevated in DR patients compared to samples from DM patients [51]. Arginine is a substrate for arginase enzymes (including arginase 1 Arg1 and arginase 2 Arg2). Overexpression of Arg2 in the diabetic retina leads to a deficiency of arginine involved in the NOS pathway, resulting in NO insufficiency and consequent vasodilator restriction and endothelial cell dysfunction, increased production of nitrogen and oxygen-responsive substances, which aggravate DR [52]. The goal of metabolomics is to discover biomarkers that provide insight into disease pathogenesis, but DR has been found to cause irreversible damage to retinopathy and is highly related to metabolic changes, so it could be used as a good technique for early detection of potential diagnostic biomarkers and treatment and prognosis management of the disease.

In the high glucose environment of the body, a large amount of reducing coenzyme II NADPH is consumed and gradually reduced to sorbitol and fructose, which leads to the decrease of reducing glutathione (GSH), an important antioxidant in the body, and the increase of reactive oxygen species (ROS) production, which enhances the oxidative stress (OS) reaction in the body [4]. OS results in the increase of advanced macromolecular glycosylation end products (AGEs), lipid peroxidation products [9]and the production of homocysteine and matrix metalloproteinase MMP2/9 [21, 22], as well as mitochondrial DNA damage which in turn reduces its transcription and expression [20]. The above series of important molecular changes may increase the expression of NADPH oxidase by activating the NF-κB signaling pathway [5]. Alternatively, the nuclear translocation and activation of Nrf2 is inhibited by the conformational modification of Keep1, so that Nrf2 cannot activate and bind to antioxidant reaction elements (ARE) and enhance the expression of related antioxidant genes [11, 12]. It may also affect mitochondrial stability by causing damage to mitochondrial structure [24]. Or the increased exudation of Cyt C promotes the apoptosis of capillary endothelial cells [20]; It could also reduce the amount of Hb by reducing erythrocytes, resulting in tissue hypoxia and increase of inflammatory factors and vascular proliferation factors [40, 41]. Eventually, it leads to retinal cell apoptosis, destruction of retinal pigment epithelium(RPE), vascular injury, increased osmotic pressure, neovascularization, macular edema, vitreous hemorrhage, retinal detachment and other results, which contribute the progression of diabetic retinopathy (DR) and even eventually lead to blindness in patients.

## Discussion

**Fig. 1:**
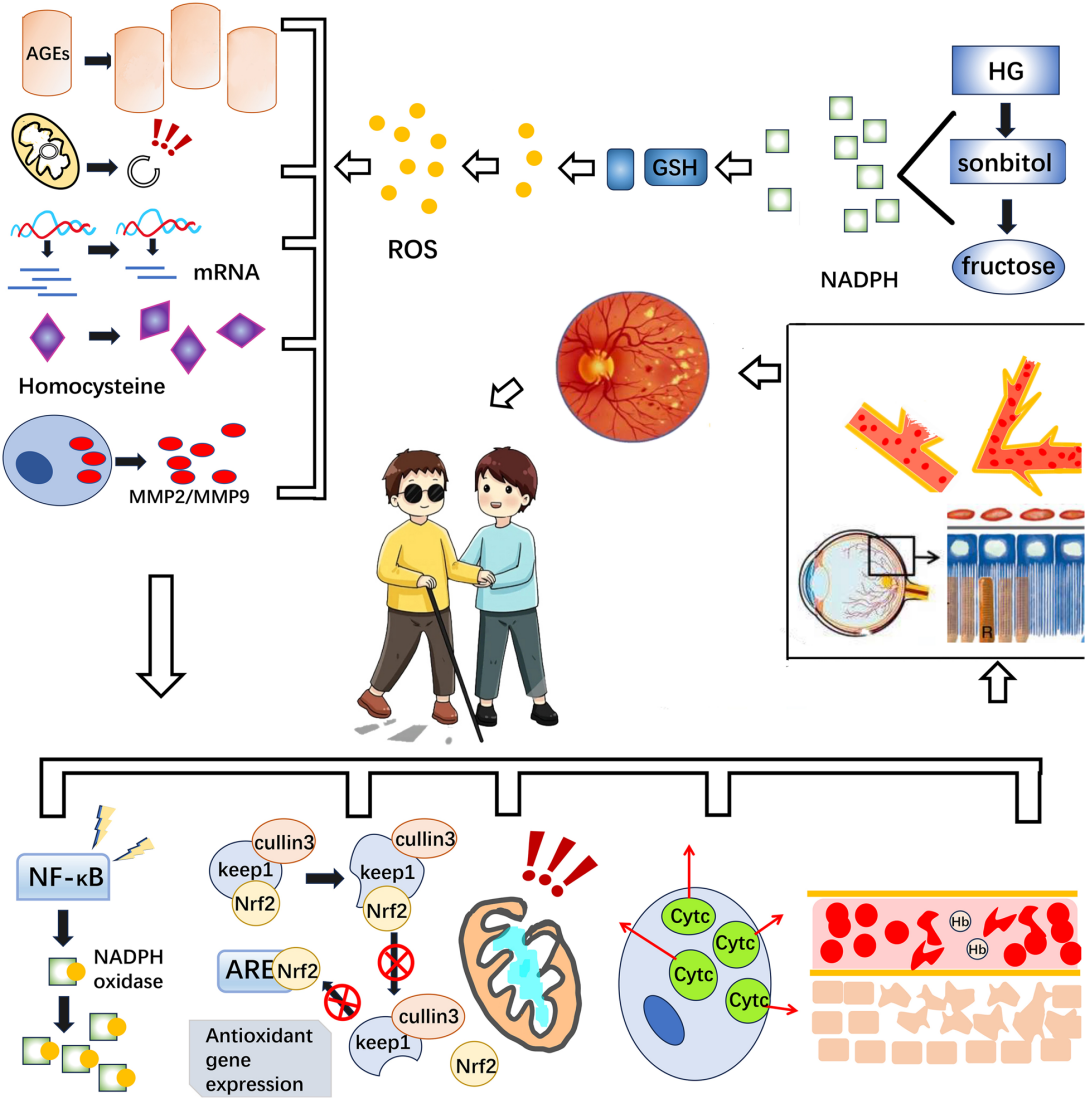
Pathways of action of relevant mechanisms in the development of DR

During the development of DR, numerous factors can interconnect and interact with each other through their own interference mechanisms to mediate a series of cellular responses, which can lead to irreversible malignant consequences. A large number of research data suggest that in the control and treatment of DR, stabilization of the mitochondrial structure, inhibition of related inflammation, modification of target genes, and prevention of oxidative stress are feasible, and the complex interactions between multiple mechanisms deserve in-depth study. The specifically relevant pathways are shown in Fig. 1. In addition, there are other signaling pathways involved in disease development. Recent studies have shown that protein kinase C-δ(PKC-δ) levels are persistently elevated in diabetic patients, and PKC-δ activation is associated with cell apoptosis. Hyperglycemia activates two independently acting pathways: Activation of PKC-δ and P38α MAPK-mediated src homology-2 domain–containing phosphatase-1(SHP-1) overexpression to inactivate platelet-derived growth factor receptor-β(PDGFR-β) and activate the NF-κB cascade to induce apoptosis of retinal pericytes and endothelial cells. It is mainly involved in the occurrence and development of early DR Lesions such as microhemangioma and acellular capillaries [53]. For the severity of DR onset and poor prognosis, early screening and diagnosis play an important role in early intervention of the disease, and both routine blood tests and differential amino acid metabolism analysis are good methods. Considering the prominent problems of frequent administration of clinical intervention therapy and the inability of long-term lasting therapeutic effects as well as serious side effects, gene therapy will become one of the hot spots for future research. Based on the characteristics of the physiological anatomy of the eye, the technical methods of vitreous cavity drug delivery and fundus examination for continuous assessment of efficacy are relatively well established, which in turn makes low-dose vector-targeted gene therapy for DR possible, but there is still a lack of relevant clinical trials. Table 1 shows the potential target molecules for intervention associated with diabetic retinopathy and their pathways of action and outcomes.

**Table 1:** Diabetic retinopathy related target molecules

Name	Related signal pathway	Function
**Nox 1/2/4/5** [7, 8]	**NF-κB**	**Enhances VEGF expression and ROS production**
**Nrf2** [11–13]	**Nrf2-ARE**	**Enhances the expression of antioxidant genes**
**HIF-1α** [41, 42]	**Increases VEGF**	**Promotes the progression of PDR**
**SHP-1** [53]	**PDGF-B/PDGFR-β** **PKC-δ** **P38αMAPK**	**Intensifies the apoptosis of peri-retinal cells and endothelial cells**
**SS31** [25]	**Decreases Drp1 and increases Mfn1/2**	**Reduces mitochondrial fragmentation and protect mitochondrial integrity**
**Homocysteine** [22]	**mmp2/9 → Cytc**	**Apoptosis of capillary cells**
**FABP4** [10]	**PPARγ → ferroptosis**	**Maintain glucose and lipid homeostasis** **Inhibit lipid peroxidation and oxidative stress**

In this review, we have outlined various pathogenic mechanisms of DR and therapeutic directions that deserve attention and in-depth exploration. As research on DR continues to progress, a deeper and more comprehensive understanding of various mechanisms that have not yet been elucidated will be gained, and various drugs or therapies will be developed to intervene and stop DR lesions at an early stage using the action characteristics of different mechanisms, which will prevent the deterioration of retinal lesions leading to blindness.

## Data Availability

Availability of data and materials is not applicable for this review article.

## References

[1] Lee R, Wong TY, Sabanayagam C (2015). Epidemiology of diabetic retinopathy, diabetic macular edema and related vision loss. Eye Vision (London, England).

[2] Tan GS, Cheung N, Simó R, Cheung GC, Wong TY (2017). Diabetic macular oedema. Lancet Diabetes Endocrinol.

[3] Domingueti CP, Dusse LM, Carvalho M, de Sousa LP, Gomes KB, Fernandes AP (2016). Diabetes mellitus: the linkage between oxidative stress, inflammation, hypercoagulability and vascular complications. J Diabetes Complications.

[4] Li C, Miao X, Li F, Wang S, Liu Q, Wang Y, Sun J (2017). Oxidative stress-related mechanisms and antioxidant therapy in diabetic retinopathy. Oxid Med Cell Longev.

[5] Wu MY, Yiang GT, Lai TT, Li CJ (2018). The oxidative stress and mitochondrial dysfunction during the pathogenesis of diabetic retinopathy. Oxid Med Cell Longev.

[6] Mima A, Qi W, Hiraoka-Yamomoto J, Park K, Matsumoto M, Kitada M, Li Q, Mizutani K, Yu E, Shimada T (2012). Retinal not systemic oxidative and inflammatory stress correlated with VEGF expression in rodent models of insulin resistance and diabetes. Invest Ophthalmol Vis Sci.

[7] Rojas M, Lemtalsi T, Toque HA, Xu Z, Fulton D, Caldwell RW, Caldwell RB (2017). NOX2-Induced Activation of Arginase and Diabetes-Induced Retinal Endothelial Cell Senescence. Antioxidants (Basel, Switzerland).

[8] Deliyanti D, Alrashdi SF, Touyz RM, Kennedy CR, Jha JC, Cooper ME, Jandeleit-Dahm KA, Wilkinson-Berka JL (2020). Nox (NADPH Oxidase) 1, Nox4, and Nox5 promote vascular permeability and neovascularization in retinopathy. Hypertension (Dallas, Tex: 1979).

[9] Chen Q, Tang L, Xin G, Li S, Ma L, Xu Y, Zhuang M, Xiong Q, Wei Z, Xing Z (2019). Oxidative stress mediated by lipid metabolism contributes to high glucose-induced senescence in retinal pigment epithelium. Free Radical Biol Med.

[10] Fan X, Xu M, Ren Q, Fan Y, Liu B, Chen J, Wang Z, Sun X (2022). Downregulation of fatty acid binding protein 4 alleviates lipid peroxidation and oxidative stress in diabetic retinopathy by regulating peroxisome proliferator-activated receptor γ-mediated ferroptosis. Bioengineered.

[11] Tebay LE, Robertson H, Durant ST, Vitale SR, Penning TM, Dinkova-Kostova AT, Hayes JD (2015). Mechanisms of activation of the transcription factor Nrf2 by redox stressors, nutrient cues, and energy status and the pathways through which it attenuates degenerative disease. Free Radical Biol Med.

[12] Liu TS, Pei YH, Peng YP, Chen J, Jiang SS, Gong JB (2014). Oscillating high glucose enhances oxidative stress and apoptosis in human coronary artery endothelial cells. J Endocrinol Invest.

[13] Tanase DM, Gosav EM, Anton MI, Floria M, Seritean Isac PN, Hurjui LL, Tarniceriu CC, Costea CF, Ciocoiu M, Rezus C (2022). Oxidative Stress and NRF2/KEAP1/ARE Pathway in Diabetic Kidney Disease (DKD): New Perspectives. Biomolecules.

[14] Feng S, Yu H, Yu Y, Geng Y, Li D, Yang C, Lv Q, Lu L, Liu T, Li G (2018). Levels of inflammatory cytokines IL-1β, IL-6, IL-8, IL-17A, and TNF-α in aqueous humour of patients with diabetic retinopathy. J Diabetes Res.

[15] Araújo RS, Santos DF, Silva GA (2018). The role of the retinal pigment epithelium and Müller cells secretome in neovascular retinal pathologies. Biochimie.

[16] Whitmore HAB, Amarnani D, O'Hare M, Delgado-Tirado S, Gonzalez-Buendia L, An M, Pedron J, Bushweller JH, Arboleda-Velasquez JF, Kim LA (2021). TNF-α signaling regulates RUNX1 function in endothelial cells. FASEB J.

[17] Valle ML, Dworshak J, Sharma A, Ibrahim AS, Al-Shabrawey M, Sharma S (2019). Inhibition of interleukin-6 trans-signaling prevents inflammation and endothelial barrier disruption in retinal endothelial cells. Exp Eye Res.

[18] Chaurasia SS, Lim RR, Parikh BH, Wey YS, Tun BB, Wong TY, Luu CD, Agrawal R, Ghosh A, Mortellaro A (2018). The NLRP3 inflammasome may contribute to pathologic neovascularization in the advanced stages of diabetic retinopathy. Sci Rep.

[19] Chen W, Zhao M, Zhao S, Lu Q, Ni L, Zou C, Lu L, Xu X, Guan H, Zheng Z (2017). Activation of the TXNIP/NLRP3 inflammasome pathway contributes to inflammation in diabetic retinopathy: a novel inhibitory effect of minocycline. Inflamma Res.

[20] Kowluru RA, Mishra M (2017). Regulation of matrix metalloproteinase in the pathogenesis of diabetic retinopathy. Prog Mol Biol Transl Sci.

[21] Kowluru RA, Mohammad G, Sahajpal N (2020). Faulty homocysteine recycling in diabetic retinopathy. Eye Vision (London, England).

[22] Kowluru RA (2020). Diabetic retinopathy: mitochondria caught in a muddle of homocysteine. J Clin Med.

[23] Zhou P, Xie W, Meng X, Zhai Y, Dong X, Zhang X, Sun G, Sun X (2019). Notoginsenoside R1 ameliorates diabetic retinopathy through PINK1-dependent activation of mitophagy. Cells.

[24] Devi TS, Somayajulu M, Kowluru RA, Singh LP (2017). TXNIP regulates mitophagy in retinal Müller cells under high-glucose conditions: implications for diabetic retinopathy. Cell Death Dis.

[25] Bhatti JS, Thamarai K, Kandimalla R, Manczak M, Yin X, Kumar S, Vijayan M, Reddy PH (2021). Mitochondria-targeted small peptide, SS31 ameliorates diabetes induced mitochondrial dynamics in Male TallyHO/JngJ mice. Mol Neurobiol.

[26] Mishra M, Kowluru RA (2015). Epigenetic modification of mitochondrial DNA in the development of diabetic retinopathy. Invest Ophthalmol Vis Sci.

[27] Kowluru RA, Santos JM, Mishra M (2013). Epigenetic modifications and diabetic retinopathy. Biomed Res Int.

[28] Shafabakhsh R, Aghadavod E, Ghayour-Mobarhan M, Ferns G, Asemi Z (2019). Role of histone modification and DNA methylation in signaling pathways involved in diabetic retinopathy. J Cell Physiol.

[29] Zorrilla-Zubilete MA, Yeste A, Quintana FJ, Toiber D, Mostoslavsky R, Silberman DM (2018). Epigenetic control of early neurodegenerative events in diabetic retinopathy by the histone deacetylase SIRT6. J Neurochem.

[30] Munk R, Panda AC, Grammatikakis I, Gorospe M, Abdelmohsen K (2017). Senescence-associated MicroRNAs. Int Rev Cell Mol Biol.

[31] Maisto R, Trotta MC, Petrillo F, Izzo S, Cuomo G, Alfano R, Hermenean A, Barcia JM, Galdiero M, Platania CBM (2020). Resolvin D1 Modulates the Intracellular VEGF-Related miRNAs of Retinal Photoreceptors Challenged With High Glucose. Front Pharmacol.

[32] Chen B, Wu L, Cao T, Zheng HM, He T (2020). MiR-221/SIRT1/Nrf2 signal axis regulates high glucose induced apoptosis in human retinal microvascular endothelial cells. BMC Ophthalmol.

[33] Li W, Jin L, Cui Y, Nie A, Xie N, Liang G (2021). Bone marrow mesenchymal stem cells-induced exosomal microRNA-486-3p protects against diabetic retinopathy through TLR4/NF-κB axis repression. J Endocrinol Invest.

[34] Xu Y, Xie SC, Ma YC (2019). Low expression of microRNA-15b promotes the proliferation of retinal capillary endothelial cells and pericytes by up-regulating VEGFA in diabetic rats. Eur Rev Med Pharmacol Sci.

[35] Fu X, Ou B (2020). miR-152/LIN28B axis modulates high-glucose-induced angiogenesis in human retinal endothelial cells via VEGF signaling. J Cell Biochem.

[36] Shan K, Liu C, Liu BH, Chen X, Dong R, Liu X, Zhang YY, Liu B, Zhang SJ, Wang JJ (2017). Circular noncoding RNA HIPK3 mediates retinal vascular dysfunction in diabetes mellitus. Circulation.

[37] Wang Y, Wang L, Guo H, Peng Y, Nie D, Mo J, Ye L (2020). Knockdown of MALAT1 attenuates high-glucose-induced angiogenesis and inflammation via endoplasmic reticulum stress in human retinal vascular endothelial cells. Biomed Pharmacother.

[38] Radhakrishnan R, Kowluru RA (2021). Long noncoding RNA MALAT1 and regulation of the antioxidant defense system in diabetic retinopathy. Diabetes.

[39] Liu P, Jia SB, Shi JM, Li WJ, Tang LS, Zhu XH, Tong P (2019). LncRNA-MALAT1 promotes neovascularization in diabetic retinopathy through regulating miR-125b/VE-cadherin axis. Biosci Rep.

[40] Lee MK, Han KD, Lee JH, Sohn SY, Jeong JS, Kim MK, Baek KH, Song KH, Kwon HS (2018). High hemoglobin levels are associated with decreased risk of diabetic retinopathy in Korean type 2 diabetes. Sci Rep.

[41] Zhang D, Lv FL, Wang GH (2018). Effects of HIF-1α on diabetic retinopathy angiogenesis and VEGF expression. Eur Rev Med Pharmacol Sci.

[42] Zhu X, Wang Y, Cheng L, Kuang H (2023). Regulation of long noncoding RNA NEAT1/miR-320a/HIF-1α competitive endogenous RNA regulatory network in diabetic retinopathy. Invest Ophthalmol Vis Sci.

[43] Wang J, Xin X, Luo W, Wang R, Wang X, Si S, Mo M, Shao B, Wang S, Shen Y (2020). Anemia and diabetic kidney disease had joint effect on diabetic retinopathy among patients with type 2 diabetes. Invest Ophthalmol Vis Sci.

[44] Complications M, Care F (2020). Standards of medical care in diabetes-2020. Diabetes Care.

[45] Ma Q, Li Y, Li P, Wang M, Wang J, Tang Z, Wang T, Luo L, Wang C, Wang T (2019). Research progress in the relationship between type 2 diabetes mellitus and intestinal flora. Biomed Pharmacother.

[46] Zhou Z, Sun B, Yu D, Zhu C (2022). Gut microbiota: an important player in type 2 diabetes mellitus. Front Cell Infect Microbiol.

[47] Jiao J, Yu H, Yao L, Li L, Yang X, Liu L (2021). Recent insights into the role of gut microbiota in diabetic retinopathy. J Inflamm Res.

[48] Scuderi G, Troiani E, Minnella AM (2021). Gut microbiome in retina health: the crucial role of the gut-retina axis. Front Microbiol.

[49] Rhee SY, Jung ES, Park HM, Jeong SJ, Kim K, Chon S, Yu SY, Woo JT, Lee CH (2018). Plasma glutamine and glutamic acid are potential biomarkers for predicting diabetic retinopathy. Metabolomics.

[50] Kritis AA, Stamoula EG, Paniskaki KA, Vavilis TD (2015). Researching glutamate - induced cytotoxicity in different cell lines: a comparative/collective analysis/study. Front Cell Neurosci.

[51] Peters KS, Rivera E, Warden C, Harlow PA, Mitchell SL, Calcutt MW, Samuels DC, Brantley MA (2022). Plasma arginine and citrulline are elevated in diabetic retinopathy. Am J Ophthalmol.

[52] Wang H, Fang J, Chen F, Sun Q, Xu X, Lin SH, Liu K (2020). Metabolomic profile of diabetic retinopathy: a GC-TOFMS-based approach using vitreous and aqueous humor. Acta Diabetol.

[53] Geraldes P, Hiraoka-Yamamoto J, Matsumoto M, Clermont A, Leitges M, Marette A, Aiello LP, Kern TS, King GL (2009). Activation of PKC-delta and SHP-1 by hyperglycemia causes vascular cell apoptosis and diabetic retinopathy. Nat Med.

